# Vestibular stimulation by 2G hypergravity modifies resynchronization in temperature rhythm in rats

**DOI:** 10.1038/s41598-020-65496-x

**Published:** 2020-06-08

**Authors:** Tristan Martin, Tristan Bonargent, Stéphane Besnard, Gaëlle Quarck, Benoit Mauvieux, Eric Pigeon, Pierre Denise, Damien Davenne

**Affiliations:** 1 UMR-S 1075 COMETE: MOBILITES “Vieillissement, Pathologies, Santé”, INSERM-Normandy University, Caen, France; 2 University, UNICAEN, ENSICAEN, LAC, 14000 Caen, France

**Keywords:** Physiology, Circadian regulation

## Abstract

Input from the light/dark (LD) cycle constitutes the primary synchronizing stimulus for the suprachiasmatic nucleus (SCN) circadian clock. However, the SCN can also be synchronized by non-photic inputs. Here, we hypothesized that the vestibular system, which detects head motion and orientation relative to gravity, may provide sensory inputs to synchronize circadian rhythmicity. We investigated the resynchronization of core temperature (Tc) circadian rhythm to a six-hour phase advance of the LD cycle (LD + 6) using hypergravity (2 G) as a vestibular stimulation in control and bilateral vestibular loss (BVL) rats. Three conditions were tested: an LD + 6 exposure alone, a series of seven 2 G pulses without LD + 6, and a series of seven one-hour 2 G pulses (once a day) following LD + 6. First, following LD + 6, sham rats exposed to 2 G pulses resynchronized earlier than BVL rats (p = 0.01), and earlier than sham rats exposed to LD + 6 alone (p = 0.002). Each 2 G pulse caused an acute drop of Tc in sham rats (−2.8 ± 0.3 °C; p < 0.001), while BVL rats remained unaffected. This confirms that the vestibular system influences chronobiological regulation and supports the hypothesis that vestibular input, like physical activity, should be considered as a potent time cue for biological rhythm synchronization, acting in synergy with the visual system.

## Introduction

Biological rhythms are mainly generated and driven by a biological clock located in the suprachiasmatic nucleus (SCN), which sends its intrinsic rhythmic outputs through neuronal and hormonal pathways^1,2^. This endogenous rhythm is around 24 hours and is thus termed a circadian rhythm. As it is usually not exactly 24 hours, the biological clock must be entrained daily to remain synchronized with external environmental time.

In most organisms, this process of entrainment occurs through the permanent exposure to light and darkness alternance induced by the Earth’s rotation^3^. The light/dark (LD) cycle information is detected by retinal sensors, whose information is mainly transmitted to the biological clock through a retinohypothalamic pathway^4–6^. Non-photic time cues have also been proven to be reliable synchronizers of the biological clock. Among them, physical activity bouts can cause modification of the daily amplitude and phase of circadian rhythms^7,8^. Although the precise mechanisms of the action of physical activity on biological rhythmicity are not known, its impact may be partly due to the activation of the vestibular system, which is highly solicited when the body is in motion.

Almost two decades ago, several studies highlighted an influence of the vestibular system on circadian rhythmicity. A vestibular stimulation through chronic hypergravity (2 G) paradigm into a centrifuge^9,10^ caused a transient loss of circadian rhythm of core temperature (Tc) for seven to 15 days. Cellular mapping studies have also demonstrated a differential c-Fos response within the SCN to hypergravity in vestibular-deficient mice, providing functional evidence of this vestibular–clock linkage^11^. Finally, only one study using knockout mice, without functional otolithic organs (het mice) and therefore with a deficient vestibular function, has demonstrated a direct effect of the vestibular system on the period of the SCN clock^12^. More recent results have confirmed this relationship between the vestibular system and biological rhythms. A total bilateral vestibular loss (BVL) after a chemical lesion^13^ caused a circadian rhythm disruption for several days before progressive recovery. Furthermore, rocking mice at 1.0 Hz impacted sleep–wake rhythmicity by increasing the time spent in NREM sleep and accelerating sleep onset, and slowed the EEG theta (6–10 Hz) frequency during the active wakefulness period. Conversely, the sleep of otoconia-deficient mice, unable to encode linear acceleration, was not affected by rocking^14^.

The vestibular system is mainly involved in gaze stabilization and postural control^15^, but it has been shown to play many roles in other functions, such as spatial learning and memory^16–18^ and autonomic regulation^19–21^. Indirect connections between vestibular nuclei and the SCN have been described^22^. During the active period (the day in humans and the dark in rodents), the vestibular system constantly captures the motion signals of the head, including gravity. The semi-circular canals and otolithic organs sense rotational and translational acceleration and deceleration^23–25^. Head movements and gravitational force changes are then daily encoded and integrated within the vestibular nuclei^26^. During rest periods, vestibular sensors are likely to be less stimulated. As the intensity of the vestibular message depends on body and head motion, it is in phase with the day and night alternance. Thus, the vestibular system could assist the visual system in entraining circadian rhythms and the sleep/wake rhythmicity.

To our knowledge, very little is known about the potent role of the vestibular system in the entrainment of circadian rhythms. To confirm and extend previous work^12^, the present study focused on vestibular stimulation as a potential synchronizer of circadian rhythms, which can accelerate the resynchronization of Tc after visually induced desynchronization. To that end, we evaluated the effect of a repeated one-hour (1 h) hypergravity 2 G pulse on the timing of circadian rhythm resynchronization after a six-hour LD phase shift in BVL and sham rats. A six-hour phase advance protocol of the LD cycle^27–29^ is known to cause a misalignment between circadian rhythms, LD cycle, and sleep/wake cycle^30,31^. Our aim was to test whether a daily 1 h hypergravity vestibular stimulation could improve the timing of Tc rhythm resynchronization of sham rats, using vestibular sensors. Conversely, we hypothesized that the Tc rhythm resynchronization pattern in BVL rats with no functional gravity receptors should not be affected by the vestibular stimulation.

## Results

### Animals’ circadian rhythms at baseline

Two groups of Long–Evans rats were used, one with total BVL and the second composed of sham-operated rats. At baseline, each rat exhibited a normal daily Tc without any difference between the two groups for acrophase (BVL 1:40 AM ± 0:38; sham 1:40 AM ± 0:31; p = 0.99), amplitude (BVL 1.0 ± 0.1 °C; sham 0.9 ± 0.2 °C; p = 0.23), and mesor (BVL 37.4 ± 0.1 °C; sham 37.3 ± 0.1 °C; p = 0.81) during control conditions lasting seven days.

Three subgroups of rats underwent three different conditions: LD + 6 condition (six-hour advance of the LD cycle), 2 G condition (1h–2 G pulse once a day in a centrifuge for seven days without phase shift of the LD), and 2 G/LD + 6 condition (daily 1h–2 G pulse for seven days after a six-hour phase advance). Each 2 G pulse occurred between 1:00 and 2:00 AM, which was the interval of time where the mean acrophase of temperature occurred at baseline.

### Resynchronization of Tc circadian rhythms

**Figure 1:**
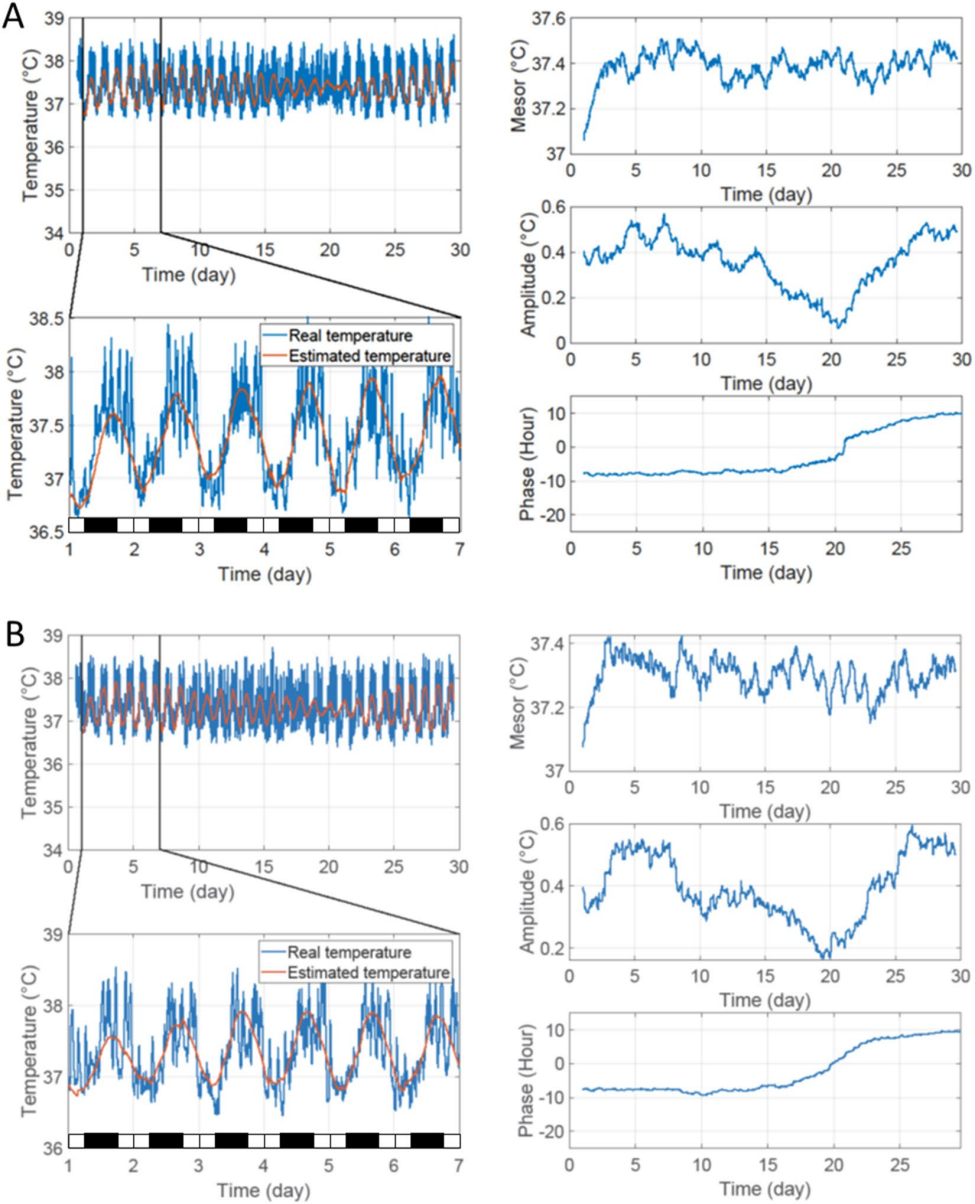
Resynchronization to a six-hour phase shift without 2 G stimulation (LD + 6 condition). Representative examples of a BVL (**A**) and a sham (**B**) rat in the LD + 6 condition. The blue line represents the recorded Tc. The orange line represents the estimated Tc from the extended COSINOR analysis. The left panels represent the entire recording of temperature (left upper panel) and a zoom on the first week of recording with estimated Tc (lower panel). The black and white bars in the left lower panel symbolize dark and light periods, respectively. The right panel (from top to bottom) represents the progressive modification of mesor (°C), amplitude (°C), and phase (hour).

**Figure 2:**
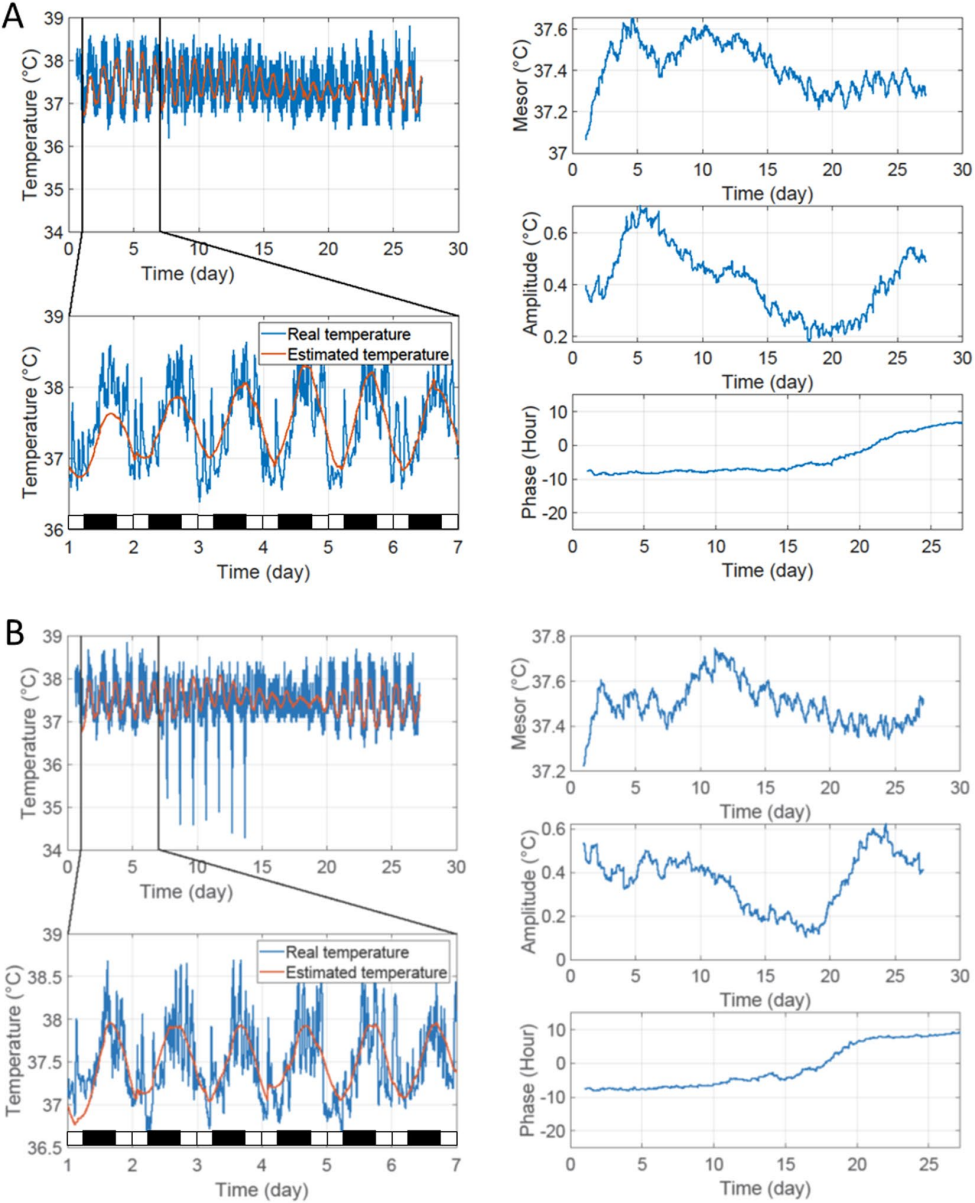
Resynchronization to a six-hour phase shift with 2 G stimulations (2 G/LD + 6 condition). Representative examples of a BVL (**A**) and a sham (**A**) rat in the 2 G/LD + 6 condition. The blue line represents the recorded Tc. The orange line represents the estimated Tc from the extended COSINOR analysis. The left panels represent the entire recording of temperature (left upper panel) and a zoom on the first week of recording with estimated Tc (lower panel). The black and white bars in the left lower panel symbolize dark and light periods, respectively. The right panel (from top to bottom) represents the progressive modification of mesor (°C), amplitude (°C), and phase (hour).

**Figure 3:**
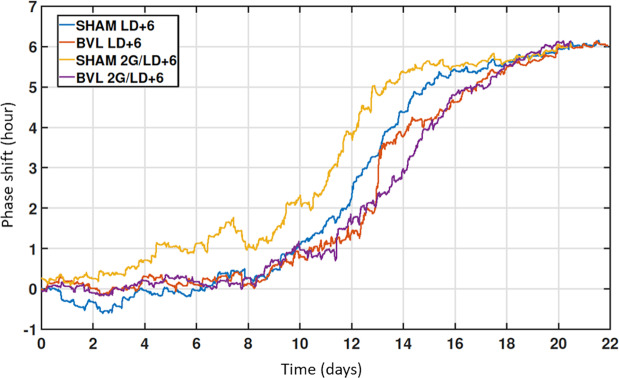
Time of resynchronization to a six-hour phase shift. Representative examples of the time of convergence of a BVL and a sham rat in both the LD + 6 and 2 G/LD + 6 conditions. Each line represents the progressive drifting of the phase to achieve resynchronization after the six-hour shift of the LD cycle.

The resynchronization pattern of Tc rhythms was characterized by the progressive drift of the phase (defined by the trough of Tc rhythm) modeled using an extended COSINOR model^32^, comparing BVL and sham groups in both LD + 6 and 2 g/LD + 6 conditions. Figures 1 and 2 illustrate a representative example of both BVL and sham groups in LD + 6 and 2 g/LD + 6, respectively. The LD + 6 condition was set up to test the effect of a six-hour phase shift on both BVL and sham rats. After a six-hour phase shift, the phase of the Tc rhythm progressively drifted from the beginning of the LD phase shift until resynchronization to the new LD, when the phase of Tc stabilized itself again. The amplitude of Tc circadian rhythms progressively decreased throughout the day and increased again when the phase was synchronized to the new LD cycle (BVL 0.95 ± 0.1; sham 0.85 ± 0.17). A significant interaction was found between groups and condition factors for time of convergence (days needed to observe resynchronization of the phase of the rhythms to the new LD cycle). In 2 G/LD + 6, sham rats resynchronized earlier than BVL rats (16.0 ± 3.1 days vs 19.6 ± 1.8 days; p = 0.01). Moreover, sham rats in 2 G/LD + 6 resynchronized earlier than sham rats in the LD + 6 condition (20.4 ± 0.8 days; p = 0.002). No significant difference was found for the delay parameters (days needed to observe the beginning of the resynchronization), meaning that Tc started its resynchronization process similarly in both BVL and sham rats in both 2 G/LD + 6 and LD + 6 conditions. All results are displayed in Table 1, and representative examples are shown in Fig. 3. The full statistical report is in Supplementary Table S2. At the end of the resynchronization period, Tc was phase advanced by 6:24 ± 0:50 hours on the overall average, with no differences between BVL or sham groups or conditions (see details in Supplementary Tables S1 and S2).

**Table 1:** Mean and SD values of the extended COSINOR method.

Parameters	Conditions	Groups	mean	SD
Time of convergence (days)	LD+6	BVL	19,6	3,0
		SHAM	20,4*	0,8
	2 G/LD+6	BVL	19,6	1,8
		SHAM	16,0*^$^	3,1
Delay (days)	LD+6	BVL	8,9	1,9
		SHAM	9,3	2,7
	2 G/LD+6	BVL	8,2	1,2
		SHAM	10,4	3,1
Tc Phase shift (h:mm)	LD+6	BVL	+6:26	0:50
		SHAM	+6:13	0:55
	2 G/LD+6	BVL	+6:15	0:57
		SHAM	+6:42	0:45

*indicates a significant difference between conditions within groups. ^$^indicates a significant difference between groups within conditions.

In summary, when BVL or sham rats were submitted to a six-hour phase shift of the LD cycle, successive 2 G pulses decreased the time needed to phase shift Tc circadian rhythms in sham rats, whereas BVL rats were unaffected by 1h–2 G pulses.

The 2 G condition was set up to test the effect of 2 G pulses without LD cycle change. BVL rats did not display any modification of their Tc circadian rhythmicity according to the extended COSINOR model in this 2 G condition. In sham rats, successive 2 G pulses led to a dampened shape of the temperature curve from the third pulse advance, leading to loss of the sinusoid shape of temperature. After the last pulse, the shape of the temperature curve progressively shifted back to the initial shape observed during baseline (Supplementary Fig. S1).

### Acute effect of 2G stimulation on Tc and locomotor activity

**Figure 4:**
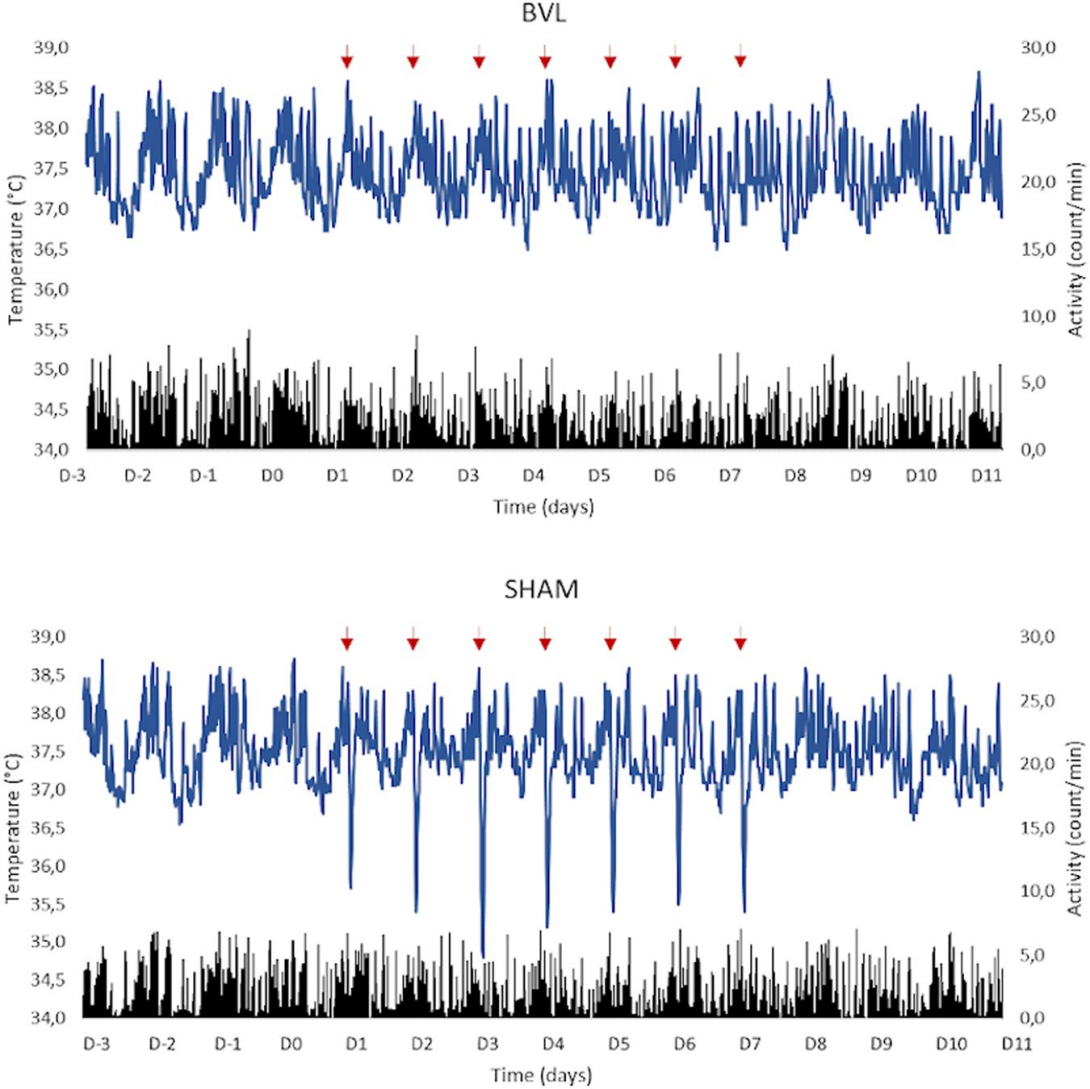
Response of BVL rats and sham rats to 2 G stimulations. Representative examples of a BVL (upper figure) and a sham rat (lower figure). The blue line represents the Tc curve, and the black bars represent locomotor activity (LMA). D-3 to D0 represents the end of the baseline recordings, and 2 G pulses started at D1. Each red arrow represents a one-hour 2 G pulse, which caused a sudden and transient drop of temperature in sham rats. For more clarity, this figure only represents three days before 2 G pulses, the seven daily 2 G pulses, and three days after 2 G pulses.

**Figure 5:**
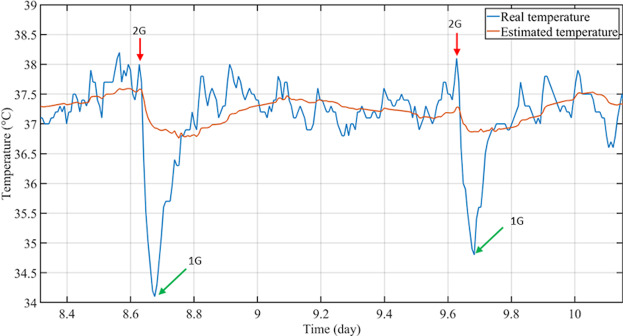
Fall of temperature in sham rats. Descriptive figure of the fall of core temperature (Tc) during the first two 2 G pulses in sham rat 16. Time is represented between the half of day 8 (first pulse) of the experiment and day 10 (0.2 days = 4.8 hours). The blue line represents the raw Tc data recorded by telemetry. The orange line represents the estimated temperature from the extended COSINOR model (see Supplementary Methods). The red arrow indicates the moment where animals were in hypergravity (2 G), and the green arrow indicates the moment where animals went back to 1 G gravity.

Each 2 G pulse (in both 2 G and 2 G/LD + 6 conditions) induced a systematic and marked fall of Tc in the sham group. This fall was not observed in BVL rats (Figs. 4 and 5).

Before each 2 G pulse, Tc level was similar in both sham (M = 37.7 ± 0.2 °C) and BVL rats (M = 37.8 ± 0.2 °C; p = 0.49). During the 1h–2 G pulses, in BVL rats, the global shape of the circadian rhythm of Tc was not drastically modified. Nonetheless, a small but significant decrease was observed (0.7 ± 0.1 °C; p < 0.001), which recovered within a few minutes at the end of the 2 G pulse (Fig. 5). Three BVL rats had a fall>1 °C (−1.2 ± 0.4 °C), whereas all others had a fall of −0.4 ± 0.3 °C.

**Figure 6:**
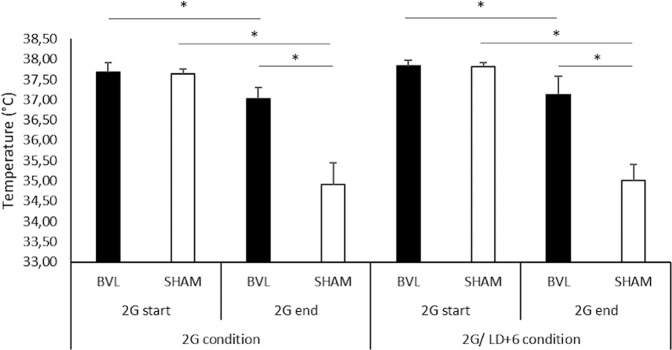
Fall of temperature caused by 2 G exposure. The histograms represent mean temperature from the seven 2 G pulses in BVL (black) and sham rats (white), in 2 G and 2 G/LD+6 conditions. “2 G start” refers to the mean Tc recorded during the five minutes just before each 2 G pulse, and “2 G end” refers to the mean Tc recorded during the five minutes at the end of the 2 G pulse.

Conversely, in sham rats, each 2 G pulse caused a significant fall in Tc (−2.8 ± 0.3 °C; p < 0.001), which was significantly greater than in BVL rats (p < 0.001; Fig. 6). These falls were consistent, with no difference observed between the seven consecutive daily pulses (p = 0.25).

During each 2 G pulse, a significant decrease of locomotor activity (LMA) was observed in both groups (p < 0.001): mean LMA fell from 3.0 ± 0.9 count/min to 2.4 ± 0.7 count/min in BVL rats, and from 2.2 ± 0.5 count/min to 0.8 ± 0.4 count/min in sham rats. The decrease was significantly higher in sham compared to BVL rats (p < 0.001).

### Mean values of Tc and LMA throughout the experiment

Mean Tc values were the same in BVL rats during the seven days of 2 G stimulations (37.34 ± 0.1 °C) and in the days of recovery (37.3 ± 0.1 °C) compared to baseline (37.4 ± 0.1 °C). In sham rats, a small but significant drop in the mean Tc (0.1 °C) was observed between baseline and the seven days when 2 G pulses occurred (37.2 ± 0.1 °C; p = 0.001). However, if the Tc values recorded during drop phases under 2 G is removed from the analysis, the mean Tc increased by 0.05 °C (p = 0.016). During the weeks following the 1 h 2 G pulses, mean Tc returned to baseline (37.3 ± 0.1 °C; see all values in Supplementary Table S1).

During baseline, the mean LMA level was significantly higher in the BVL group (1.9 ± 0.2 count/min) than in the sham group (1.5 ± 0.3 count/min; p < 0.001). During the seven days of 2 G pulses, mean LMA level was significantly decreased in both the BVL group (1.6 ± 0.24 count/min; p = 0.001) and the sham group (1.1 ± 0.2 count/min; p < 0.001), with a larger decrease in the latter (p < 0.001). This decrease of mean LMA remained significant in both groups even when data recorded during each 1 h 2 G pulse were removed (BVL 1.6 ± 0.2 count/min; sham 1.1 ± 0.02 count/min; p < 0.001). In the days of recovery, LMA remained lower than in the baseline condition in both BVL (1.7 + 0.3 count/min; p < 0.001) and sham groups (1.3 + 0.2 count/min; p = 0.003), again with a larger decrease in the latter (p < 0.001).

LMA and Tc were thus not influenced in the same way by 2 G gravity. The effect of the vestibular system on circadian rhythm is not solely attributable to a masking effect of LMA.

## Discussion

The aim of this study was to explore the effect of a vestibular stimulation through a 1 h 2 G pulse applied during seven days on the restoration of temperature circadian rhythm after a six-hour shift of the LD cycle. The main results show that stimulating the vestibular system through short periods of 2 G hypergravity affects the circadian rhythm of Tc after a six-hour LD cycle phase shift. Sham rats exposed to 1 h 2 G pulses exhibited a higher rate of resynchronization to the new LD cycle than did BVL rats deprived of gravity receptors, and earlier than sham rats exposed to a six-hour advance of the LD cycle only.

In this study, we evaluated how Tc resynchronizes after a six-hour advance of the LD cycle with and without 2 G stimulation. We focused on Tc since it is a reliable reflection of biological clock activity, thermogenic neurons in the hypothalamic areas being connected to SCN^33^. Tc has been used as a marker of rhythmicity in several studies using desynchronization or hypergravity paradigms^9,34^.

Hypergravity 1 h 2 G pulses led to a sharp drop in the Tc signal, making classic methods of analysis unable to estimate rhythmicity. The first challenge was to develop a specific model to analyze the Tc daily variation and resynchronization by avoiding the induced-hypergravity temperature drop (see Methods). This temperature decrease, already observed in previous studies using hypergravity^9,10,35^, was a good indicator of the effect of vestibular stimulation on Tc. This will be discussed further, although mechanisms were specifically studied in the current research. Furthermore, LMA was highly disrupted throughout the experiment compared to Tc, making it impossible to apply the developed model of analysis, so we focused on LMA mean level but not on LMA rhythm resynchronization. LMA was not considered a marker of rhythmicity in the current study, but measured to control a potential masking effect on Tc to ensure that the former is not responsible for the latter’s pattern^36^.

Concerning the resynchronization of Tc after a six-hour advance of the LD cycle only (LD + 6 condition), the phase of Tc circadian rhythms became out of phase with the LD cycle and started to progressively drift from its initial value until resynchronization, without differences between BVL or sham rats. The six-hour advance of the LD cycle also caused a progressive lowering of the amplitude of Tc until resynchronization of the phase. This is in accordance with earlier studies, which have shown that this lower amplitude of circadian rhythms at the physiological level after desynchronization was due both internal desynchronization within the SCN^37,38^ and desynchronization between the SCN and peripheral clocks^27^. When applying a daily 1 h 2 G stimulation after the six-hour advance of the LD cycle (2 G/LD + 6 condition), sham rats shortened the total time of resynchronization of the Tc phase compared to other sham rats with no 2 G stimulation. Furthermore, BVL rats lacking vestibular gravity sensors also receiving a daily 1 h 2 G stimulation were not affected by these repeated 2 G pulses and resynchronized in the same way as rats (BVL or sham) submitted to a six-hour advance of the LD cycle only.

The current study confirms and extends previous work in the field^9,12^ by showing that stimulating the vestibular system through short periods of hypergravity could act as a signal affecting circadian rhythm resynchronization. Several studies have shown that chronic hypergravity paradigm into a centrifuge (1.5–2 G)^35,39,40^ caused a transient loss of Tc circadian rhythm for seven to 15 days, but without clear evidence of vestibular system involvement. However, the precise mechanism through which vestibular stimulation affects circadian rhythms is not known. Among them, a mechanism to be discussed is that the vestibular system could influence the circadian rhythms through non-photic inputs to the SCN itself, affecting its activity. The links between the vestibular system and Tc circadian rhythm were firstly clearly demonstrated through the disruption of circadian rhythms in wild-type rodents under chronic 2 G hypergravity environments, whereas circadian rhythms in knockout het mice (without otolithic organs) were not affected^9,10^. Moreover, by demonstrating that het mice present alterations in their intrinsic period characteristic and response to changes of constant-light environment, Fuller and Fuller^12^ hypothesized that vestibular (otolith organ) signaling sub-serves activity-based non-photic regulation of the clock. More recently, a translational rocking motion induced sleep and wake EEG characteristics in mice with otolithic organs^14^, whereas mice without otolithic organs were not affected by such a motion^14^. Moreover, downstream NREM sleep-promoting neurotensinergic neurons have been identified in the medial vestibular nucleus^41^.

A pathway between the vestibular nuclei and the posterior hypothalamus, which plays an important role in biological rhythmicity, has been described^42^. A reciprocal connection between the vestibular nuclei and orexin neurons of the lateral hypothalamus has also been suggested, with orexin neurons playing an important role in the sleep–wake cycle^43^. Furthermore, the vestibular nuclei are indirectly connected with the suprachiasmatic nuclei via the intergeniculate leaflet (IGL)^22^. The IGL is involved in the transmission of both photic and non-photic inputs towards the SCN and probably mediates the effect of physical activity on circadian cycles^44,45^. Such non-photic entrainment properties have been shown with physical activity bouts in rats^46,47^. Because visual and vestibular afferences to the IGL projecting to the SCN are strongly entangled anatomically^22^, vestibular input could thus be complementary and in synergy with the visual system to modulate biological rhythms and sleep/wake cycle regulation. It is possible that both light and vestibular information interacted during the resynchronization process in the current study. For this reason, we set up both LD + 6 (LD phase shift; no vestibular stimulation) and 2 G (no LD shift; vestibular stimulation) conditions. As described above, BVL and sham rats resynchronized in the same way in the LD + 6 condition. In the 2 G condition, no phase shift of the Tc circadian rhythm was observed at the end of the week of 2 G stimulation. This may imply that the vestibular system, despite involvement in circadian rhythm entrainment in this study, cannot overwhelm the effect of light. If the true nature of this entrainment by physical activity is still unknown, the vestibular system could be at least partially involved. The existing neuronal pathway between vestibular nuclei and IGL may convey information for movement linked to arousal, spontaneous motor activity, and physical activity to the SCN.

Another unexplored, but reliable hypothesis, is that transient hypothermia related to 2 G might be strongly implicated in the induced-hypergravity earlier resynchronization. The acute 2 G vestibular stimulation caused an initial large fall of Tc for one hour in sham rats, followed by a rise to the initial level within hours after the 2 G pulse. This effect was reproducible all along the week where 1 h 2 G pulses were applied. Moreover, the effect was similar in all sham rats, whether they were exposed to the six-hour phase shift (2 G/LD + 6 condition) or not (2 G condition). Conversely, the Tc of BVL rats was barely affected by daily 2 G pulses. The present study confirms previous results, where stimulation of the vestibular receptors in normal mice was responsible for the fall of Tc, whereas Tc was not affected in knockout het mice during 2 G centrifugation^9,10^. It also confirmed that the vestibular system has a strong link with thermoregulatory mechanisms^35,39,48^. The slight decrease of Tc observed in BVL rats in the present study was similar to the one observed in knockout het mice in the studies cited above. The precise mechanism is still unknown and the current study cannot elucidate it. Previous studies hypothesized that this phenomenon would be an inadequate metabolic response to 2 G^49^ when head acceleration is not in the normal range of sensibility of the vestibular system^13^. Fuller *et al*.^50^ postulated that both changes in body fluids distribution (increase in heat loss in peripheral and acute suppression of heat production at central level) and altered convection mechanism may contribute to Tc changes in altered gravity, even though no consensus still exists^49^. Decrease in heat production through the brown adipose tissue or increase in tail blood flow has also been observed during stimulus (hypergravity or spinning inducing motion sickness) inducing hypothermia^51,52^. Recent results support a new hypothesis which involves the autonomic nervous response. In this preliminary report hypothermia induced by hypergravity exposure would be due to the activation of glutamatergic Vglut2 neurons in the vestibular nuclei in mice^53^.

Other studies have shown that induced hypothermia leads to increased SCN activity^54,55^. There is a lack of evidence that the hypothermia itself modulates the synchronization. Hypothermia signals could modify the circadian system through a feedback loop, but this has yet to be confirmed. Further studies are needed to separate the different mechanisms related to hypergravity and to hypothermia. This can be done by inhibiting hypothermia episodes during 2 G, or using strong transient hypothermia, cold or heat exposure.

No modification of the mean Tc all along the week of 2 G stimulation was observed in either of the two groups. While a small but significant drop in mean Tc was observed in sham rats between baseline and the week of 2 G stimulation, it was due to a masking effect of the abnormal fall of temperature during the 2 G pulses. The mean LMA level was significantly reduced in both groups, even apart from 2 G pulses. Previous studies using knockout het mice have shown a significant decrease in the mean LMA level during chronic 2 G gravity without incidence on Tc^9^. This supports the hypothesis that LMA and Tc are not influenced in the same way by 2 G gravity and that the effect of the vestibular system on circadian rhythm is not attributable to a masking effect of LMA^13^. Other sensory systems (mainly somatosensory and proprioception) or mechanics due to the increase of body mass could influence LMA during 2 G centrifugation^9,10^.

The current study has several limitations. First, we only used 2 G hypergravity, as in major studies in the field. Smaller intensities (1.25–1.5 G) may have been as efficient as 2 G to entrain circadian rhythms but less influenced LMA patterns. Investigating the appropriate number and timing of pulses to enhance resynchronization is also necessary. Finally, we focused on Tc daily variation to confirm what previous studies in the field have observed. Further studies including more circadian parameters (hormones such as melatonin and cortisol) and EEG recording to study the sleep–wake cycle are needed to confirm the relationship between vestibular and circadian systems.

Nonetheless, this study shows that the vestibular system relates to the circadian timing system. Vestibular stimulation by repeated 2 G stimulation accelerated the resynchronization process of Tc to a phase advance of the LD cycle. If light is still considered the major entraining agent of biological rhythms, the vestibular system is necessarily put into play by detecting the behavioral activity. The results show an application in promoting physical challenge to the vestibular system while practicing, which would thus help reinforce circadian rhythm resynchronization after jet lag, or in populations with circadian and sleep disorders.

## Methods

### Ethical approval

Experiments were carried out in accordance with the European Communities Council Directive 2010/63/UE and French law. The protocol was approved by the animal ethical committee: Comité National de Réflexion Ethique sur l’Expérimentation Animale (CENOXEMA) in April 2012 (registration 0412–02).

### Animals

Male Long–Evans rats (n = 48; 300–350 g, Janvier, France) were individually housed under constant temperature conditions (21 ± 1 °C), with a 12-hour light/12-hour dark cycle (light on from 08:00 AM to 8:00 PM). Food and water were available ad libitum. Under anesthesia, each rat was implanted intraperitoneally with a telemetric device to continuously record the Tc (°C) and LMA (count/min) by actimetry. The rats were randomized into two groups. The first group underwent BVL by injection with arsanilate^56^ (BVL group, n = 24); the second group consisted of sham-operated rats by injection of saline (sham group, n = 24) and was used as the control group. All rats were kept in convalescence for one month under 12-hour light/12-hour dark (LD, light on from 8:00 AM to 8:00 PM). Seven rats were excluded from analysis due to abnormal telemetric data recording or incomplete vestibular lesion in the case of BVL rats.

### Surgical procedure to record Tc and LMA

A TA-F40 Data Science International^®^ (St Paul, Minnesota, USA) device was used to measure Tc and LMA, the latter defined by all horizontal linear motion, throughout the experiment without interruption. The device is a cylindrical implant with a 12-month lifetime (duration of battery operation). Implantation of the sensor was carried out as described by Data Science International^®^. The sensor was implanted intra-abdominally after midline laparotomy under isoflurane anesthesia. Animals were kept in convalescence for 15 days after the implementation of a telemetric sensor. An intraperitoneal injection of 2 mL antibiotic (amoxicillin–clavulanic acid: Augmentin^®^) and 2 mL analgesic (paracetamol) was given once per day for three days to prevent nociception.

### Vestibular lesion procedure

The chemical model of a vestibular lesion by trans-tympanic arsanilate injection has already been described in our previous study^13^. It leads to a selective lesion of vestibular hair cells without any damage to the external ear tract, Eustachian tubes, oropharynx, cranial nerve VIII, or Scarpa’s ganglion, with no diffusion of arsanilate through the peripheral tissues or blood, or through the sheath of cranial nerve VIII up to the brainstem^56^. Each rat in the BVL group received a single bilateral dose (0.1 mL/30 mg) of sodium arsanilate (Sigma-Aldrich©) dissolved in 0.9% saline solution under volatile anesthesia (2% isoflurane) in oxygen (flow rate of 2 L/min). The injection was through the anterior part of the tympanum using a 1-mL syringe (needle diameter: 0.8 mm), and arsanilate was deposited into the middle ear cavity^56^. The sham group received a single bilateral injection of 0.9% saline solution (0.1 mL) by the same route. Each rat then received one dose of paracetamol (Prodafalgan, Merck^©^) that was intraperitoneally injected (1 mL/25 mg) twice per day for two days to decrease nociception due to the tympanic lesion. BVL syndrome was assessed each week after lesion using a validated clinical vestibular scale^57^. The complete procedure is described in the study of Vignaux *et al*.^56^ and has been linked to complete loss of vestibular function up to three months after chemical lesion.

### Protocol

**Figure 7:**
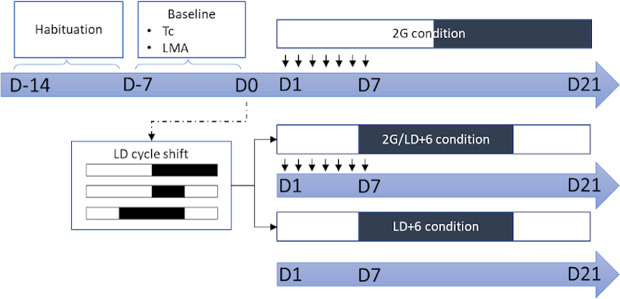
Experimental procedure. Habituation: Seven days where rats lived in their cages, placed in the centrifuge. Baseline: recording Tc and LMA before LD shift. D0: 6 h advance of the LD cycle during the night for 2 G/LD+6 and LD+6 conditions only. Each black arrow indicates a 1 h 2 G pulse.

Figure 7 summarizes the experimental protocol. At the end of the convalescence, rats were divided into three subgroups (n = 8 BVL and n = 8 sham), which underwent three different conditions: being exposed to a six-hour shift of the LD cycle (LD + 6) induced by the shortening of the dark period^27^, being exposed to 1 h 2 G pulses once a day for seven days (2 G), and a combined condition (2 G/LD + 6). During the experiment, eight rats (four BVL and four controls) were individually housed in the four-arm centrifuge (two cages per compartmented arm; one rat per cage) in a 12/12 LD cycle (300 lux; light from 8:00 AM to 8:00 PM). After a week of habituation living in the centrifuge, Tc and LMA were measured continuously for six days before LD shift (baseline).

Rats of the LD + 6 and 2 G/LD + 6 conditions were submitted to a six-hour phase advance (light on from 02:00 AM to 2:00 PM), accomplished by advancing the time of lights-on, leading to one short six-hour night. In the 2 G/LD + 6 condition, the first 2 G pulse was applied in the night following the six-hour LD cycle shift. The timing of the pulse was determined according to research on exercise and circadian rhythms. Using 3 h exercise bouts starting in the first hours after night onset showed entrainment in rats^47^. This corresponds to the acrophase of Tc, where the highest activity phase occurs^58,59^, at the time when the vestibular system is normally more solicited. The daily 1 h 2 G pulse was thus applied in the interval of the acrophase of temperature at baseline, meaning at 1:00 AM (CT 17). Seven 2 G pulses of one hour were given in total. The centrifuge was programmed to reach 2 G within five minutes. After the week of stimulation, the rats were kept in the new LD cycle for 15 days. Once the first eight rats finished the experiment, eight others were placed in the centrifuge. The cages were cleaned once a week, four to six hours before lights-out, to avoid disturbance during the vestibular stimulation period, except during the week of 2 G stimulation.

### Analysis of daily rhythms

Due to unstable period and amplitude changes in sham rats (because of either LD shift, 2 G pulses, or both) preventing comparison between groups, an original technique was used to consider the stimuli and other possible disturbances. This method allowed us to model Tc data despite the presence of an acute drop of Tc in sham rats disturbing the sinusoidal pattern. Data from TA-F40 were first converted in five-minute mean bouts. Tc data obtained from each rat during the control conditions were used to calculate the different parameters of the daily rhythms (amplitude, acrophase, and mesor) using an extended COSINOR analysis, which allowed determination of the best fit of a combined 24-hour period cosine function. The extended COSINOR model is based on the classical model of the following form:

$$Y({t}_{i})=M+A{\rm{\cos }}(\omega {t}_{i}+\phi )$$

where ϕ, A, and M represent the daily acrophase (peak time), the peak to trough amplitude, and mesor (mean level), respectively. $\omega {t}_{i}$ corresponds to the fixed 24-hour period of the rhythm. Each period was validated at p < 0.05. The extended COSINOR model adds two terms. The first corresponds to the effect of stimuli or other sources of disturbances, and the second to the noise in the data. These additions improve the accuracy of the results.

The time required for complete resynchronization of Tc circadian rhythms following the six-hour phase advance was then calculated for each rat, as well as the time of convergence, by identifying the changes of its 24-hour periodic circadian pattern. To do that, we used an extended COSINOR model consisting of a recursive least square algorithm. It can estimate non-stationary parameters of a model, here the COSINOR model^60^.

Two methods were used to estimate the dynamic of the phase. The first estimates the convergence time required for complete resynchronization after the stimulus with regard to the phase. Briefly, this method computes a threshold from the derivative in the interval $[{t}_{1},{t}_{2}]$, with ${t}_{2}$ corresponding to the moment after the time of convergence of the parameters and ${t}_{1}$ the time of the stimulus. The value of the threshold is the maximum of the derivative plus an arbitrary value of 40% chosen to be coherent with all the rodents present in the experience. The convergence time corresponds to the last instant the derivative is above the threshold minus the time of the stimulus. The second method estimates the “delay” time the rats take to start their phase shift. The idea is to match a model on the filtered estimated phase. All details about the two models are available in Bonargent *et al*.^32^ and provided in the Supplementary Methods. The phase shift of the acrophase of Tc at the end of the experiment was thus calculated for each rat.

### Statistical analysis for group comparisons

Tc circadian rhythms of the BVL and sham groups in baseline were first compared using a t-test to ensure that the groups were comparable. Then, the falls in Tc during 2 G pulses were compared using a three-way RM ANOVA (group x condition x time), with BVL and sham as a group factor, with a condition factor (2 G, 2 G/LD+6) and a time factor (baseline, 2 G week and recovery). The same procedure was used to compare mean Tc and LMA. Resynchronization parameters during LD+6 and 2 G/LD+6 conditions (time of convergence, delay, and Tc phase shift) were compared using a two-way MANOVA (group x condition). Post hoc tests (Tukey) were performed if a significant interaction was detected. The significance threshold was set at p < 0.05.

The datasets generated during and/or analysed during the current study are available from the corresponding author on reasonable request.

## Supplementary information

Supplementary information.
