# Supplementary material for: Vestibular stimulation by 2G hypergravity modifies resynchronization in temperature rhythm in rats
Source: Sci Rep. 2020 Jun 8;10:9216. doi: 10.1038/s41598-020-65496-x (PMC7280278; doi:10.1038/s41598-020-65496-x)
Supplement: Supplementary file 1 — Supplementary information. [file 41598_2020_65496_MOESM1_ESM.docx]

Supplemental Material for:

**Vestibular stimulation by 2G hypergravity modifies resynchronization in temperature rhythm in rats**

**Tristan Martin^1*^, Tristan Bonargent^2^, Stéphane Besnard^1^, Gaëlle Quarck^1^, Benoit Mauvieux^1^, Eric Pigeon^2^, Pierre Denise^1^, Damien Davenne^1*^**

^1^Normandie Univ, UNICAEN, INSERM, COMETE, 14000 Caen, France

^2^Univ, UNICAEN, ENSICAEN, LAC, 14000 Caen, France

This supplemental material for initial submission contains the following information:

Supplemental Figure

Supplemental Tables

Supplemental Methods: estimation of circadian rhythm parameters

# Supplemental Figures


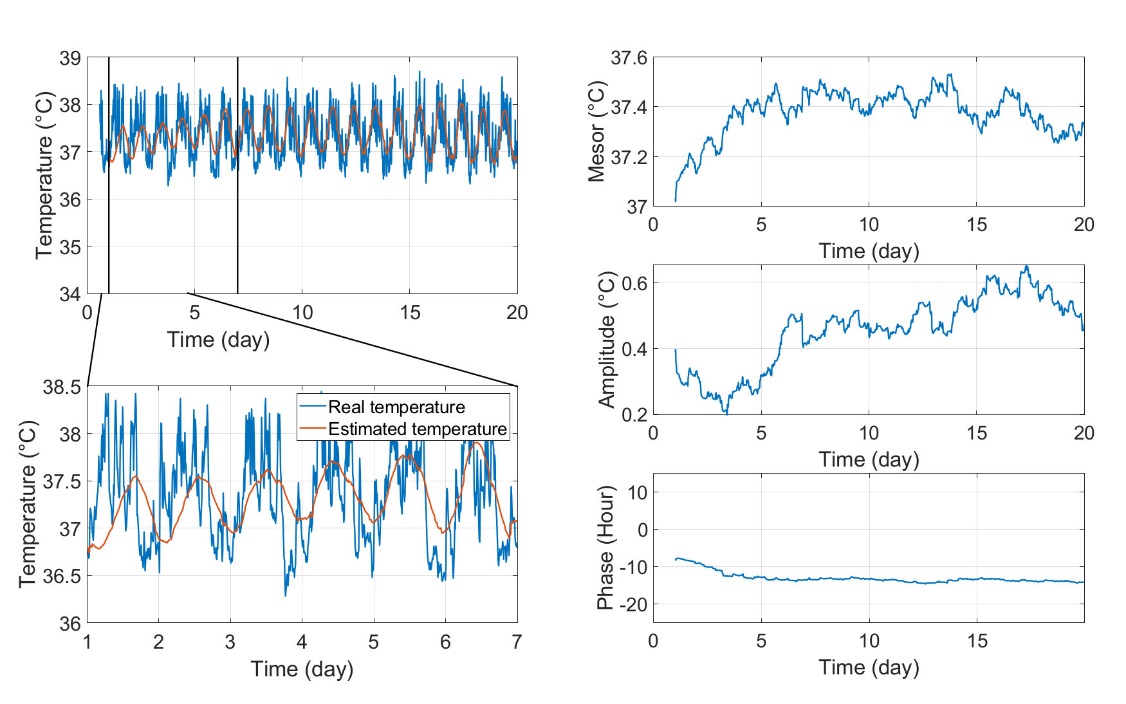

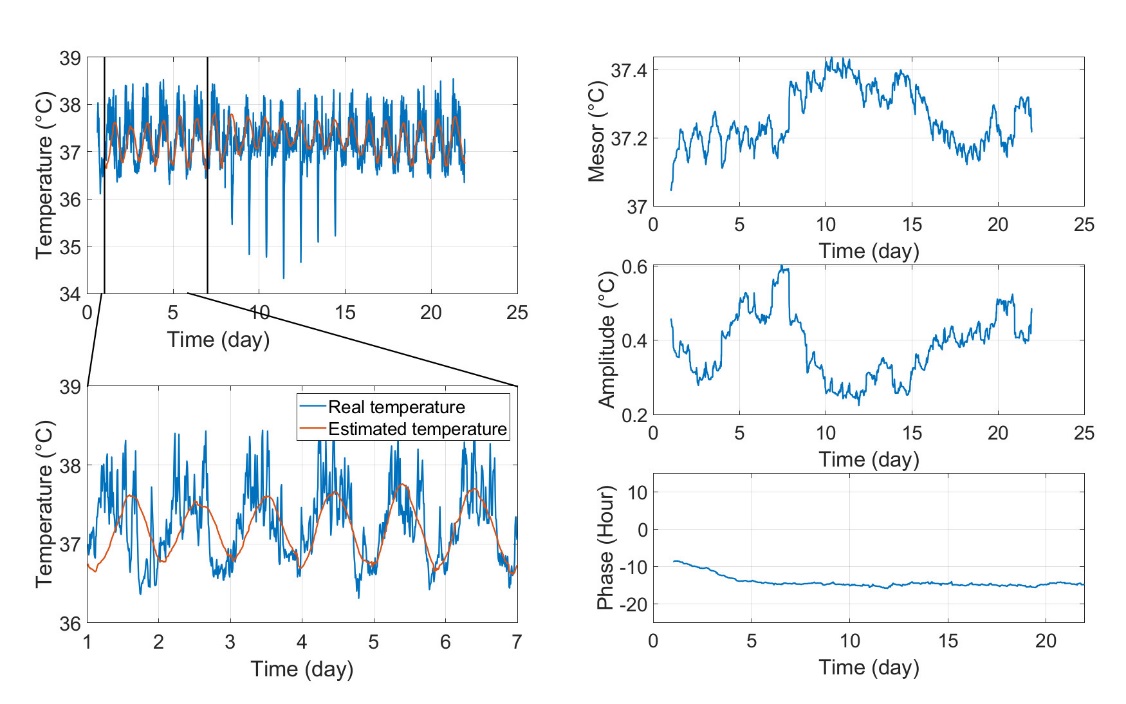


**B**

**A**

**Supplementary Figure S1**. **Representative examples of a BVL (A) and a sham (B) rat in 2G condition.** Blue line represents the recorded Tc. Orange line represents the estimated Tc from the extended COSINOR analysis. Left panels represent the entire recording of temperature (left upper panel) and a zoom on the first week of recording before 2G pulses with the estimated Tc (left lower panel). Right panel (from top to bottom) represents the progressive modification of Mesor (°C), amplitude (°C) and phase (Hour).

# Supplemental tables

**Supplementary Table S1. Mean values for Tc and LMA**.

Mean values for Tc and LMA in rats subjected to 2G pulses at baseline, during the week where 2G pulses were given (2G week) and during the recovery period after 2G pulses series.

|  | baseline | 2G week | recovery |
| --- | --- | --- | --- |
| BVL Tc (°C) |  |  |  |
| M | 37,4 | 37,3 | 37,3 |
| SD | 0,1 | 0,1 | 0,1 |
| SHAM Tc (°C) |  |  |  |
| M | 37,3 | 37,2 | 37,3 |
| SD | 0,1 | 0,1 | 0,1 |
| BVL LMA (count/min) |  |  |  |
| M | 1,9 | 1,7 | 1,7 |
| sd | 0,2 | 0,2 | 0,3 |
| Sham LMA (count/min) |  |  |  |
| M | 1,5 | 1,1 | 1,3 |
| sd | 0,3 | 0,2 | 0,2 |

**Supplementary Table S2. MANOVA and Post Hoc Tests results for extended COSINOR parameters**.

Significant threshold has been set at p<0.05 (indicated by *)

|  |  | df | Mean square | F | Sig. |
| --- | --- | --- | --- | --- | --- |
| condition * groups | Convergence time | 1, 23 | 33,429 | 5,892 | 0,023 |
|  | Delay | 1, 23 | 5,266 | 0,931 | 0,345 |
|  | Phase shift | 1, 23 | 0,693 | 0,917 | 0,348 |
| condition | Convergence time | 1, 23 | 31,467 | 5,546 | 0,027 |
|  | delay | 1, 23 | 0,361 | 0,064 | 0,803 |
|  | Phase shift | 1, 23 | 0,146 | 0,194 | 0,664 |
| groups | Convergence time | 1, 23 | 13,557 | 2,389 | 0,136 |
|  | Delay | 1, 23 | 11,051 | 1,954 | 0,175 |
|  | Phase shift | 1, 23 | 0,087 | 0,115 | 0,737 |

| Groups x condition | | | | | | | |
| --- | --- | --- | --- | --- | --- | --- | --- |
| Parameters | Conditions | Groups | *p* |  | Groups | Conditions | *p* |
| Convergence | LD+6 vs 2G/LD+6 | BVL | 0,960 |  | BVL vs SHAM | LD+6 | 0,531 |
|  |  | SHAM | 0,002* |  |  | 2G/LD+6 | 0,011* |
| Delay | LD+6 vs 2G/LD+6 | BVL | 0,626 |  | BVL vs SHAM | LD+6 | 0,758 |
|  |  | SHAM | 0,389 |  |  | 2G/LD+6 | 0,115 |
| Phase shift | LD+6 vs 2G/LD+6 | BVL | 0,723 |  | BVL vs SHAM | LD+6 | 0,660 |
|  |  | SHAM | 0,324 |  |  | 2G/LD+6 | 0,378 |

# Supplementary methods

ESTIMATION OF CIRCADIAN RHYTHM PARAMETERS

The aim of this section is to characterize the adaptation of the rats faces to a perturbation. The parameters of the COSINOR model are estimated with a recursive least square algorithm. In fact, the phase is the best physical quantity to estimate the shift to the new rhythm. Two methods of quantification of the shift will be exposed after a description of the model and the algorithm procedure.

## The extended COSINOR model

The algorithm used for the estimation of circadian rhythm parameters is a recursive least square algorithm. It can estimate non-stationary parameters of a model, here the COSINOR model [1]. The idea of the algorithm is to minimize the quadratic error between the measured temperature and the calculated model.

The classic COSINOR model is described by the following equation.

$\Theta\left( t \right)=M+A\cos(\omega t+\varphi)$ 

with $\Theta(t)$ the temperature,$M$ the mesor, $A$ the amplitude, $\varphi$ the phase, ω the frequency (rad/s) and t the discrete time.

The identification objective is to provide a consistent estimation of $M$, $A$ and $\varphi$ with the hypothesis that ω is known and corresponds to a period of 24 hours.

The standard model [2] is extended to take perturbations and noise into account that are related to the movements made by the rat during the day and the centrifugation. This lead to the following extended model.

$\Theta\left( t \right)=M+A\cos\left( \omega t+\varphi\right)+B\left( q^{-1} \right)u\left( t \right)+C\left( q^{-1} \right)e(t)$ 

With $B\left( q^{-1} \right)=b_{0}+b_{1}q^{-1}+b_{2}q^{-2}+\ldots+b_{n_{b}}q^{-n_{b}}$, $C\left( q^{-1} \right)=1+C_{1}q^{-1}+C_{2}q^{-2}+\ldots+C_{n_{c}}q^{-n_{c}}$, $n_{b}$ and $n_{c}$ respectively the order of $B\left( q^{-1} \right)$ and $C\left( q^{-1} \right)$, $u\left( t \right)$the input that represents the perturbations due to the centrifugation and $e(t)$ a white noise.

The input u(t) is composed of seven centered steps of one hour corresponding to one centrifugation experiment per day.

The COSINOR model (1) can be linearized as follow:

$\Theta\left( t \right)=M+\alpha\cos\left( \omega t \right)+\rho\sin\left( \omega t \right)+B\left( q^{-1} \right)u\left( t \right)+C\left( q^{-1} \right)e(t)$

 $\Theta\left( t \right)=\phi^{T}\left( t \right)\theta\left( t \right)+e(t)$ 

With $\alpha=A\cos(\varphi)$ and $\rho=A\sin(\varphi)$

The vector of parameters $\theta\left( t \right)$ and the observations vector $\phi\left( t \right)$ are defined by

$\theta\left( t \right)=[M \alpha\rho b_{0} b_{1}\ldots c_{1} c_{2}\ldots]$ 

$\phi^{T}\left( t \right)=[1,\cos\left( \omega t \right), \sin\left( \omega t \right), u\left( t \right), \ldots, u\left( t - n_{b} \right), e\left( t - 1 \right), \ldots, e\left( t-n_{c} \right)]$

## Definitions of vectors

As the white noise is not accessible, the vector $\phi(t)$ is replaced by the vector $\hat{\phi}(t)$ changing the noise $e\left( t-i \right)$ by the a posteriori error $\varepsilon\left( t-i/ t-i \right)$ respecting the equation (5).

$\varepsilon\left( t-i/ t-i \right)=y\left( t-i \right)-\hat{\phi}^{T}(t-i)\hat{\theta}(t-i)$ 

The estimated parameters vector $\hat{\theta}(t)$ and the vector $\hat{\phi}(t)$ are defined in the equations (6) which respect the precedent modifications.

$\hat{\theta}(t) =[\hat{M} \hat{\alpha} \hat{\rho} \hat{b_{0}} \hat{b_{1}}\ldots\hat{c_{1}} \hat{c_{2}}\ldots]$

 $\hat{\phi}^{T}\left( t \right)=[1, \cos\left( \omega t \right), \sin\left( \omega t \right), u\left( t \right), \ldots, u\left( t- n_{b} \right), \ldots, \varepsilon\left( t-1/t-1 \right), \ldots, \varepsilon\left( t-n_{c}/t-n_{c} \right) ]$ 

## An adaptation of the recursive least square algorithm

The selected algorithm is a robust modification of the recursive least square algorithm [3]. It integrates a freeze of identification that permits to avoid the drift of the estimated parameters and hence to ensure a consistent convergence even if the persistent excitation condition is not fulfilled. The system of equations (7) represents the algorithm [4].

$\left\{ \begin{aligned} \varepsilon\left( t / t-1 \right)=y\left( t \right)-\hat{\phi}^{T}\left( t \right)\hat{\theta}(t-1) \\ \delta\left( \varepsilon\left( t / t-1 \right) \right)=\left\{ \begin{aligned} 0 if |\varepsilon\left( t / t-1 \right)|<\gamma_{w} \\ \frac{{1-\gamma}_{w}}{|\varepsilon\left( t / t-1 \right)|} otherwise \end{aligned} \right. \\ \Gamma\left( t \right)=\frac{\delta\left( \varepsilon\left( t/t-1 \right) \right)F\left( t \right)\hat{\phi}\left( t \right)}{1+\delta\left( \varepsilon\left( t/t-1 \right) \right)\hat{\phi}^{T}\left( t \right)F\left( t \right)\hat{\phi}\left( t \right)} \\ \hat{\theta}\left( t \right)=\hat{\theta}\left( t-1 \right)+\Gamma\left( t \right)\varepsilon\left( t/t-1 \right) \\ F\left( t+1 \right)=\left( 1-\beta\right)\left( I_{n}-F\left( t \right)\hat{\phi}^{T}\left( t \right) \right)F\left( t \right)+\beta F_{0} \end{aligned} \right.$ 

with $n=3+n_{b}+n_{c}+1$, $\beta\in\mathbb{R}^{+}$ relatively small that respect $0<\beta<1$ and $\gamma_{w}$ an infimum of the imperfection of the model.

The validation of this algorithm has been confirmed thanks to the autocorrelation rate of the residuals corresponding to the a posteriori error $\varepsilon\left( t / t \right)$ [3]. A level of significance of 3% has been chosen which means that the autocorrelation rate must be under a threshold of value $2.17N$ to respect the condition, with $N$ the number of samples.

ESTIMATION OF THE DYNAMIC

Two methods have been used to estimate the dynamic of the phase. The first estimates the convergence time required for complete resynchronization after the stimulus related to the phase while the second estimates the bandwidth and the delay, which respectively represent the speed of resynchronization and the time the rats take to start their phase shift

## Time of convergence

The time of convergence is calculated from the derivative of the identified phase which can be estimated thanks to a discrete Luenberger observer [5]. For that purpose, let us consider the following state-space model.

$\left\{ \begin{aligned} \dot{x}\left( t \right)=Ax\left( t \right) \\ y\left( t \right)=Cx\left( t \right) \end{aligned} \right.$ 

with$A=\left[ \begin{matrix} 1 & T_{e} \\ 0 & 1 \end{matrix} \right]$, $C=\left[ \begin{matrix} 1 & 0 \end{matrix} \right], x(t)=\left( \begin{matrix} x_{1}(t) & x_{2}(t) \end{matrix} \right)^{T}\in\mathbb{R}^{2}$ is the state, $y(t)=x_{1}(t\mathbb{)\in R}$ the phase and $T_{e}$ the sampling period. Please note that $x_{2}(t)$ denotes the first derivative of $y(t)$ and hence represents the signal to be estimated.

Based on the model (8), the Luenberger observer that provides a consistent estimation $\hat{x}(t){=\left( \begin{matrix} \hat{x}_{1}(t) & \hat{x}_{2}(t) \end{matrix} \right)}^{T}$ of $x(t)$ is given by

$\dot{\hat{x}}\left( t+1 \right)=A\hat{x}\left( t \right)+Bu\left( t \right)-K(\hat{y}\left( t \right)-y\left( t \right))$ 

where the gain $K\in\mathbb{R}^{2\times1}$ is calculated to adjust the eigen values of the matrix $\left( A-KC \right)$in the stability domain and to ensure a compromise between the speed of convergence and the observer sensitivity to the noise measurement.

A threshold is calculated from the derivative in the interval$[t_{1},t_{2}]$, with $t_{1}$ which corresponds to the moment after the time of convergence of the parameters and $t_{2}$ the time of the stimulus. The value of the threshold is the maximum of the derivative plus an arbitrary value of 40% chosen to be coherent with all the rodents present in the experience.

The time of convergence corresponds to the last instant the derivative is above the threshold by removing the time of the stimulus. Fig S2 represents the idea.

## Bandwidth and delay

The second method estimates an adaptation speed of rats and a time the rats take to start their phase shift. The idea is to match a model on the filtered estimated phase. For more clarity, bandwidth has been removed from the results in the main article. The model is given by the following delayed second order transfer function

$G\left( s \right)=K_{p}\frac{{-T}_{z}+s}{1+\frac{2\varsigma}{\omega_{0}}s+{(\frac{s}{\omega_{0}})}^{2}}e^{-T_{d}s}$ 

with $K_{p}$ the static gain, $\varsigma$ the damping, $\omega_{o}$ the bandwidth that is related to the adaptation speed, $T_{d}$ the delay that is related to the time taken before the shift and $T_{z}$ the zero.

To compute the model an input must be defined. It depends on the type of experimentation of the rat.


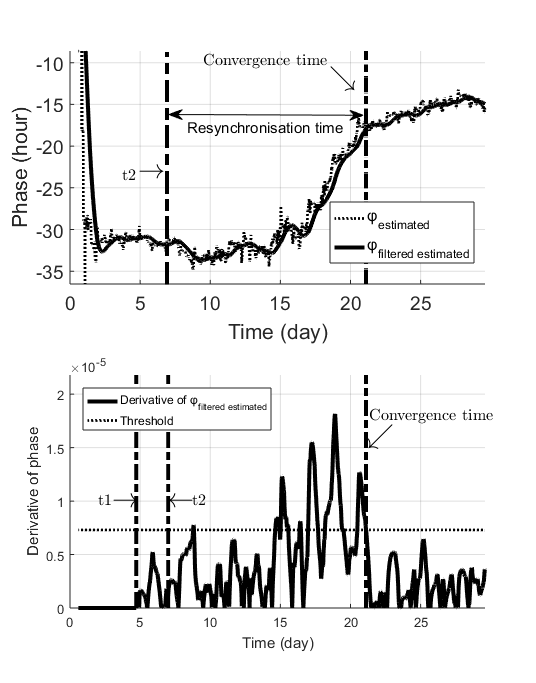


Supplementary Figure S2 Estimation of the time of convergence

#### Jet lag (SHAM and BVL) and Jet lag plus 2G (only BVL)

For this group, the model is preceded during all the experiment without the first samples before the instant $t_{1}$. The input is fixed to 0 before the instant of jet lag $t_{2}$and 1 hour after, as shows in the Fig. S3.


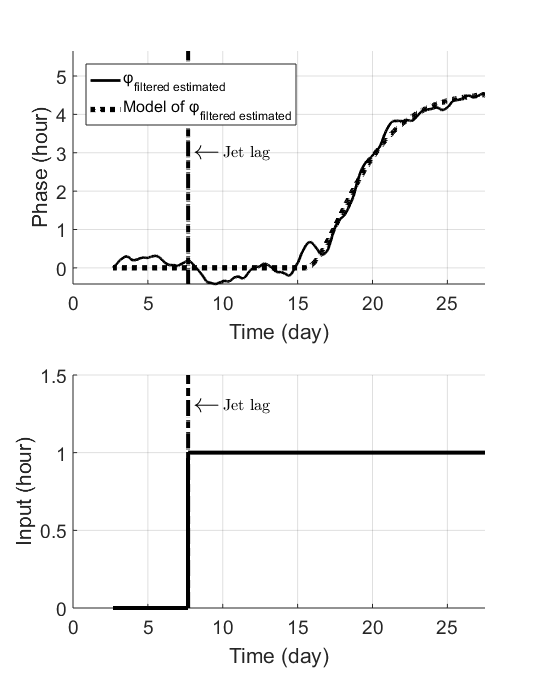


Supplementary Figure S3 Estimation of a model: Jet lag (SHAM and BVL) and Jet lag plus 2G (only BVL)

#### 2G (only SHAM) and Jet lag plus 2G (only SHAM)

For this group, the interesting part is the variation of the phase during the recovery. The modelization begins seven days after the first pulse. The input is a step set to one hour, seven days after the first stimulus as shows in the fig. S4.


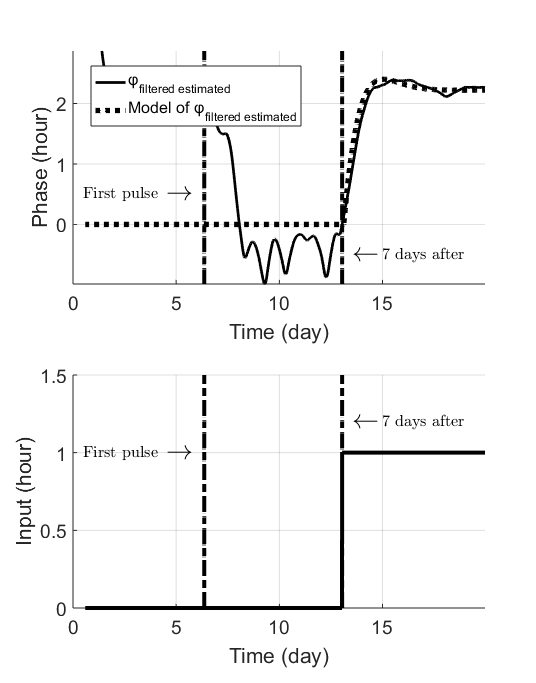


Supplementary Figure S4 Estimation of a model: 2G (only SHAM) and Jet lag plus 2G (only SHAM)

#### 2G (only BVL)

As the variation of the phase doesn't not represent a system, no model has been estimated for this group.

##### References

R. Refinetti, G. Cornélissen, et F. Halberg, « Procedures for numerical analysis of circadian rhythms », Biol. Rhythm Res., vol. 38, no 4, p. 275‑325, août 2007.

E. Pigeon et al., « Identification of circadian rhythm », in 2016 24th Mediterranean Conference on Control and Automation (MED), 2016, p. 791‑796.

L. Ljung, System Identification: Theory for the User. Prentice Hall PTR, 1999.

M. Pouliquen, E. Pigeon, et O. Gehan, « Identification Scheme for Hammerstein Output Error Models With Bounded Noise », IEEE Trans. Autom. Control, vol. 61, no 2, p. 550‑555, févr. 2016.

D. Luenberger, « An introduction to observers », IEEE Trans. Autom. Control, vol. 16, no 6, p. 596‑602, déc. 1971.
